# Bilateral Progressive Visual Loss in an Epileptic, Mentally Retarded Boy

**DOI:** 10.4103/0974-9233.75892

**Published:** 2011

**Authors:** Silvana Guerriero, Michele Vetrugno, Lorenza Ciracì, Lucia Artuso, Rosa Dell’Aglio, Vittoria Petruzzella

**Affiliations:** Department of Ophthalmology-Otolaringology, University of Bari, Bari, Italy; 1 Department of Medical Biochemistry, Biology and Physics, University of Bari, Bari, Italy; 2 CNR Institute of Biomembrane and Bioenergetic (IBBE), Bari, Italy

**Keywords:** Epilepsy, Leber’s Hereditary Optic Neuropathy, Migraine, Vision Loss

## Abstract

Leber’s hereditary optic neuropathy (LHON) is a maternally inherited, monosymptomatic disorder, characterized by severe central vision loss and optic atrophy that most frequently affects young men. The classic LHON phenotype is associated to three mitochondrial DNA mutations, mostly homoplasmic, in the Mt-ND4, Mt-ND6, and Mt-ND1 genes, encoding for complex I subunits of the mitochondrial respiratory chain. Rare cases have been described in the literature in association with variable central nervous system involvement in a syndromic form called LHON ‘*plus*.’ In the present study, we report the case of a 16-year-old boy with the 3460/ND1 mutation who presented with epilepsy, migraine, and mental retardation as non-ophthalmic features. We also investigated his relatives who all had the 3460/ND1 mutation.

## INTRODUCTION

We present evidence of a novel form of Leber’s hereditary optic neuropathy (LHON) *plus* phenotype characterized by an association of epilepsy, migraine, and mental retardation with the main ocular features in a 16-year-old boy with the 3460/ND1 mutation. We also investigated his relatives who were all positive for the 3460/ND1 mutation.

## CASE REPORT

A 16-year-old boy from Southern Italy presented to the ophthalmology department (OD) with a history of sub-acute, painless, and rapidly progressive bilateral vision loss. One month prior to presentation, he had noticed impaired sight in his right eye, and within a few days he could only see shadows. One week after the loss of sight in his right eye, the same symptoms occurred in his left eye.

The patient was born full term and was the first of three siblings. The patient had epilepsy that developed at 7 years of age, with recurrent tonic clonic seizures that were under good control with Phenobarbital. He also suffered from mild mental retardation, psychiatric disorders, and migraine. He had no history of smoking or alcohol abuse. He denied any illicit drug use. The family history was significant for mental retardation and hyperactivity in the mother and siblings. At the time of presentation, the patient appeared to be a healthy, well-developed boy but with clear signs of anxiety. His oral temperature was 36°C. His pulse had a regular rhythm, with a rate of 70 beats/min and his blood pressure was 105/60 mmHg. His lungs were clear at auscultation, with normal respiratory effort. The first heart sound (S1) and second heart sound (S2) were normal. The abdomen was soft and not tender. The peripheral arterial pulses in the upper and lower extremities were normal.

**Figure 1 and 2:**
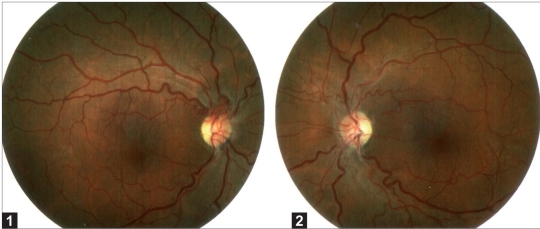
Fundus examination at onset of the visual loss, showing a hyperaemic optic nerve and telangectatic and tortuous peripapillary vessels

His best corrected visual acuity (BCVA) was light perception in the right eye and 20/800 in the left eye. On slit-lamp examination, the cornea, anterior chamber, and lens were normal; the pupils were isochoric; there was a relative afferent pupillary defect OD; fundus examination showed a hyperemic optic nerve and telangectatic and tortuous peripapillary vessels [Figures 1 and 2]. On the suspicion of bilateral optic neuropathy, the patient was admitted to our department, where he underwent: (1) laboratory investigations, including a complete blood count and basic metabolic panel, autoantibodies (antinuclear antibodies, anti-double-strand deoxyribonucleic acid (DNA) (anti-DNA) antibodies, anti Estractable Nucleart Antigens (anti-ENA), Antineutrophil Cytoplasmic Antibodies (ANCA), Neuro Myelitis Optica-IgG, serology for Lyme disease, syphilis, herpes virus, and cytomegalovirus that were all negative; (2) visual field examination in the left eye, showing a wide central scotoma. Visual field examination of the right eye could not be performed due to the profound vision loss; (3) magnetic resonance imaging of the skull and orbit, which were normal; (4) cerebrospinal fluid examination, showing no cells and no oligoclonal bands; (5) visual evoked potential, revealing signs of a conduction disturbance in the optic nerves; (6) retinal angiography, which demonstrated a normal fluorescence of the optic disk and tortuous retinal vessels bilaterally; (7) electroencephalography, which showed abnormalities in the frontal and temporal areas; (8) electromyography, which was normal; and (9) neurological examination, which revealed that Cranial nerves II-XII were intact, as well as a normal tendon reflex. Bilateral optic neuritis was diagnosed and the patient was treated with methylprednisolone at doses of 1 gr/day for three days and 500 mg/day for 3 days, but no visual improvement was observed. On the suspicion of mitochondrial neuropathy, after informed consent, total DNA samples were extracted from peripheral blood lymphocytes by a standard procedure. LHON mutations were screened by restriction fragment length polymorphism polymerase chain reaction analysis using the enzyme BstXI for T14484C, the enzyme S*fa*NI for G11778A, and the enzyme H*ga*I for G3460A. Sequencing of specific mitochondrial DNA (mtDNA) regions was also performed by ABI PRISM 310 analyzer (*Applied Biosystems Inc., Foster City, CA, USA*). Restriction fragment length polymorphism polymerase chain reaction analysis for all the three primary LHON mutations were homoplasmic for the G3460A/ND1 mutation. Sequencing of the mitochondrial marker regions of the proband to test the presence of the 12308 (tRNALeu), but not of the 10394 (ND3) variant, allowed us to assign our proband to haplogroup U. Additional Cytochrome *b* (Cyt*b*) gene sequence analysis revealed the presence of the only T15693C Cyt*b* variant which defined the sub-haplogroup U4.

**Figure 3:**
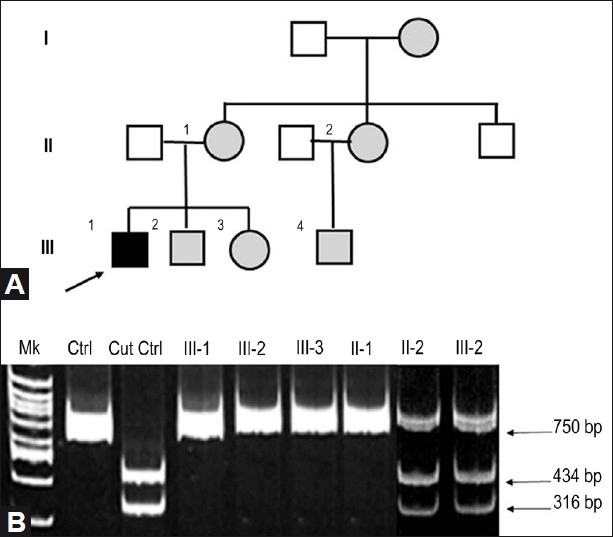
(A) Pedigree of a LHON Plus Family: The proband is shown in black; open symbols represent asymptomatic subjects; Gray symbols represent the affected family members who underwent clinical evaluation. (B) Densitometric analysis of the digestion products

We also investigated his relatives: his 38-year-old mother [Figure 3A, subject II-1] presented with BCVA of 20/20 and mental retardation; his 13-year-old brother [Figure 3B, subject III-2] presented BCVA of 20/25 OD and 20/20 OS, mental retardation, hyperactivity, and frequent vomiting. His 9-year-old sister [Figure 3A, subject III-3] presented with BCVA of 20/40 in both eyes, mental retardation, hyperactivity, and frequent vomiting. His 35-year-old aunt [Figure 3A, subject II-2] presented with BCVA of 20/20 and mild mental retardation. The aunt’s son was a 12-year-old boy [Figure 3A, subject III-4] with BCVA of 20/20, mild mental retardation, and hyperactivity. Fundus examination was normal for patient’s mother, the aunt, and cousin; the optic nerve head was hyperemic in the siblings. The family history was also significant for cardiomyopathy (grandmother) [Figure 3A, subject I-1]. Cardiomyopathy and mental retardation are often linked to chromosomal abnormalities; indeed, we excluded a dysmorphic syndrome by clinical examination. The cardiologic examination including the electrocardiogram and echocardiogram were performed and were well within normal limits. There child was not an offspring of a consanguineous marriage. We believe, therefore, that this case provides further support to the notion of heterogeneity in LHON.

After informed consent was obtained, the total DNA samples were extracted from peripheral blood lymphocytes of the family members by standard procedure. Restiction Fragment Length Polymorphism (RFLP)-Polymerase Chain Reaction (PCR) analysis for all the three primary LHON mutations were homoplasmic for the G3460A/ND1 mutation in subjects II-1, III-2, III-3, whereas in subjects II-2 and III-4 it was present at 40% of mutated 3460/ND1 *vs* total mtDNA as assessed by densitometric analysis of the digestion products [Figure 3B]. The presence of G3460A mutation was confirmed by direct sequencing of the ND1 region.

## DISCUSSION

The hyperemic optic nerve and telangectatic and tortuous peripapillary vessels led us to suspect a mitochondrial neuropathy, in particular LHON. Vascular changes in LHON have been noted since the initial descriptions.^1^ Typically in the active phase, there is an increased arteriovenous shunting in the telangectatic vascular bed, with dilatation and tortuosity of the peripapillary arterioles.^1^ The optic disk appears hyperaemic in the active phase, and becomes pale and atrophic, whereas the microangiopathic signs disappear as the vision loss stabilizes.^2^

LHON is a maternally inherited disorder characterized by bilateral visual loss that typically progresses over weeks to months without pain, until bilateral central scotomas and dyschromatopsia develop and profound visual loss occurs. The disease most frequently affects young males, with a mean age of onset in the mid-20s, although the range is extremely broad.^1^ Common features of the acute phase include peripapillary microangiopathy and optic disc atrophy.^2^ The classic LHON phenotype is associated to three mtDNA mutations: G3460A, G11778A, and T14484C, in the ND1, ND4 and ND6 genes, respectively, all encoding for complex I subunits of the mitochondrial respiratory chain. The LHON pathogenic mutations do not express a similar pathogenic potential: the severity of the biochemical defect in LHON shows a clear gradient from the 3460/ND1 (most severe), to the 14484/ND6 (mildest), passing through the intermediate features of the 11778/ND4 mutation.^2^ The 3640/ND1 mutation is the most penetrant when homoplasmic, presents with less gender difference and has a very low rate of visual recovery. The G3460A/ND1 mutation affects a more restricted number of LHON patients and is frequently heteroplasmic.^3^ The G11778A/ND4 and T14484C/ND6 changes are the most frequent LHON mutations and are mostly homoplasmic; spontaneous visual recovery often occurs with the T14484C/ND6 mutation.

Certain environmental factors may exert an influence on LHON penetrance, triggering pathological features in previously unaffected mutation carriers. Smoking, alcohol abuse,^4^ exposure to toxins,^5^ uncontrolled diabetes,^6^ head trauma,^7^ pharmaceutical agents such as ethambutol^8^ and anti-retroviral therapy are risk factors.

It has been reported that the 3460/ND1 mutation, when associated with specific cytochrome b (cytb) variants, specifically 15773/cytb alone or in combination with 15693/cytb, may contribute to the clinical expression of a syndromic form of optic atrophy referred to as LHON ‘*plus*.’^9^ This syndromic form may include variable central nervous system (CNS) involvement such as lesions of the basal ganglia, a Leigh-like syndrome, cerebellar atrophy, migraine, epilepsy and peripheral neuropathy,^1^ cardiac involvement with conduction abnormalities,^10^ skeletal deformities^11^ and myoclonus.^9^–^12^

The clinical and genetic findings in the patient we describe led us to make a diagnosis of LHON ‘plus’ associated with the mtDNA 3460/ND1 mutation. The clinical features were characterized by acute optic neuropathy in both eyes, with acute severe sequential visual loss, a rapid onset of optic atrophy, as well as some extra-ocular features, namely epilepsy, migraine and mental retardation.

The current therapeutic approaches to prevent optic atrophy in LHON are limited and work on the premise of correcting the metabolic defect through drug-based therapies. Therapeutic attempts in LHON have recently been based on the use of Coenzyme Q10 (CoQ_10_) or its short chain derivative (Idebenone),^13^ in order to restore the electronic flow in the mitochondrial respiratory chain and increase the antioxidant defenses. The alpha-2 receptor agonist brimonidine has recently been studied due to its neuroprotective proprieties that occur by maintaining the mitochondrial membrane potential. This agent is currently used in glaucoma and whether or not such a medication is a suitable candidate for LHON therapy, particularly in the attempt to rescue the second eye is yet to be deterined in controlled studies. It is also important to advise LHON patients not to smoke or drink alcohol and to avoid exposure to a variety of toxins.

After diagnosis, our patient was treated with Idebenone (100 mg b.i.d.)^14^ and Brimonidine eye drops for two years, but he showed no improvement despite these medications.

## CONCLUSIONS

We report a novel case of LHON plus disease in an Italian patient carrying the 3460/ND1 mutation (homoplasmic) in which the classic clinical features of the disease are associated to seizures, mild mental retardation, psychiatric disorders and migraine.

The LHON plus phenotype in our cases may relate to the synergic role of mtDNA variants (presence of the 12308 (tRNALeu) and of the T15693C cyt*b* variant). These genetic variants probably influence the expression of the disease, leading to a complex involvement of the CNS in which optic neuropathy is only the ‘tip of the iceberg.’
